# Effects of Probiotic Supplementation on Gut Microbiota and Fecal Metabolome in Autism Spectrum Disorders: A Secondary Analysis of a Randomized Clinical Trial in Preschoolers

**DOI:** 10.3390/metabo16040262

**Published:** 2026-04-13

**Authors:** Letizia Guiducci, Luca Laghi, Nicolò Dellarosa, Paola Mastromarino, Margherita Prosperi, Filippo Muratori, Sara Calderoni

**Affiliations:** 1 Institute of Clinical Physiology IFC-CNR, Via Giuseppe Moruzzi 1, 56124 Pisa, Italy; 2 Department of Agricultural and Food Sciences, University of Bologna, 47521 Cesena, Italy; l.laghi@unibo.it (L.L.); nicolo.dellarosa@gmail.com (N.D.); 3 Section of Microbiology, Department of Public Health and Infectious Diseases, Sapienza University, 00185 Rome, Italy; paola.mastromarino@uniroma1.it; 4 UFSMIA Valdera-Alta Val di Cecina, Azienda USL Toscana Nord Ovest, 56128 Pisa, Italy; margherita.prosperi@uslnordovest.toscana.it; 5 Stella Maris Mediterraneo Foundation, 85032 Chiaromonte, Italy; filippo.muratori@fsm.unipi.it; 6 IRCCS Fondazione Stella Maris, 56100 Pisa, Italy; sara.calderoni@fsm.unipi.it; 7 Department of Clinical and Experimental Medicine, University of Pisa, 56100 Pisa, Italy

**Keywords:** autism spectrum disorders, gastrointestinal symptomatology, microbiota, fecal metabolome, probiotics

## Abstract

Background/Objectives: Recently, a randomized clinical trial evaluated whether a six-month probiotic administration could reduce symptom severity in preschool children with Autism Spectrum Disorders (ASD), with (GI) or without (NGI) gastrointestinal symptoms. Significant positive changes were observed only in NGI children. A second explorative study on children prior to intervention identified a fecal metabolome fingerprint associated with ASD severity. Building on these findings, the present study aimed to assess whether metabolomics could monitor changes in ASD severity following probiotic administration using a subset of samples from the same trial. Second, this study aimed to identify fecal metabolites to be monitored in children to predict whether their autism severity may decrease after probiotic or placebo treatment. Methods: Evaluations of the fecal metabolome and microbiota could be completed on 57 children before and after a double-blind administration of a probiotic mixture or a placebo. Results: In NGI children the probiotic was found to influence the concentration of the amino acids aspartate, leucine, tryptophan, and valine, together with nicotinate and the short chain fatty acids acetate, butyrate, isobutyrate, and propionate. Lactobacilli and *Sutterella* showed significant changes in response to probiotic administration (*p* < 0.05). Acetate, 4-hydroxyphenyl, galactose, proline, and tyramine were identified as key fecal metabolites for prediction purposes. Conclusions: The present exploratory analysis, despite the small sample size, suggests that fecal metabolomics may provide a useful approach for monitoring and potentially for predicting changes in ASD severity following probiotics administration.

## 1. Introduction

Autism Spectrum Disorders (ASD, autism) are a group of heterogeneous neurodevelopmental disorders with a prevalence rate of 1.15% in Italy. The core symptoms of ASD include difficulties with social interaction and communication, as well as patterns of restricted, repetitive behaviors and sensory processing abnormalities [1].

A wide range of psychiatric [2] and medical [3] comorbidities are common in individuals with ASD, among which gastrointestinal (GI) problems (e.g., constipation, diarrhea, and abdominal pain) are consistently reported as more prevalent in children with ASD than in typically developing (TD) peers (for a recent systematic review, see the work by Leaderer et al. [4]). In this context, the possible contributing role of the gut microbiome in ASD pathogenesis has been analyzed, and several research investigations highlighted an alteration in the microbiome composition of ASD subjects compared to age-matched TD subjects [5]. In particular, reduced gut microbiota diversity [6,7] and imbalance in gut microbial composition [8,9] have been detected in autistic people compared with TD peers. Still, no specific and univocal microbiome signature for ASD has been recognized.

The rationale for probiotic use in autism forms part of this scenario, since probiotic administration in ASD strives to restore the healthy balance of the altered gut microbiota [10]. Several studies on individuals with ASD explored the potential of therapeutic interventions with probiotic supplementation, reporting in some cases beneficial effects not only for specific GI symptoms, but also for core ASD impairments [11,12,13].

Recently, a randomized clinical trial (RCT) [14] was conducted in 85 preschool children with Autism Spectrum Disorder (ASD), with (GI) or without (NGI) gastrointestinal symptoms, to determine whether a six-month administration of the De Simone Formulation (DSF) probiotic mixture could reduce their GI symptoms and ASD severity, assessed using the Total Autism Diagnostic Observation Schedule (ADOS). The primary analysis showed no significant changes in ADOS scores when all children were considered as a single group. In contrast, an exploratory secondary analysis stratified by GI status revealed a significant reduction in ADOS scores in NGI children following probiotic administration [15].

During the same trial, two fecal samples per child were collected, one before and one after the intervention, in a subgroup of 80 children. These samples allowed Laghi et al. [16] to perform a subsequent analysis on baseline samples showing that ASD severity was associated with the water-soluble fecal metabolome, fecal microbiota, and levels of the intestinal inflammation biomarker calprotectin. Concerning fecal metabolome, they did this by selecting molecules whose concentration significantly differed between children with high and low severity of ASD, corresponding to high and low ADOS scores. Then, they described the trends underlying this reduced portion of the overall fecal metabolome by calculating a robust principal component analysis (rPCA) model. They found that the very first principal component (PC1) was sensitive to ASD, so that children with low and high ADOS scores appeared at high and low PC1 scores, respectively.

Principal component analysis is commonly used to reduce the number of highly correlated variables, allowing underlying trends to become more apparent. When a link between the reduced variables and the feature under investigation is established, the model can be effectively employed as a prediction tool on samples that have been newly analyzed. Laghi et al. applied this principle to the connection between the fecal metabolome and autism severity by projecting the metabolome of the children with intermediate ADOS scores on the model. Coherently with the existence of a link be-tween autism severity and fecal metabolome, the PC1 score of these children appeared as intermediate between high and low ADOS children.

Prompted by the highlighted link between autism severity and the fecal metabolome on baseline samples by Laghi et al. [16], the present work extends the exploration of the fecal metabolome to the samples collected after the treatment, to investigate the potential impact of the DSF probiotic mixture administration. No new clinical recruitment or laboratory sample collection was performed.

Two sections describe changes in fecal microbiota and metabolome associated with improvement or worsening of ASD symptoms in preschool children after six months.

The first part of the study is focused on discovering the metabolites that, independently of the treatment, marked a shift in the subjects’ conditions. The second part outlines the benefits of the DSF probiotic mixture on the microbiota of children with ASD, highlighting changes in the relative abundance of gut species and the modulation of key biomarker concentrations that may activate specific pathways following probiotic treatment.

## 2. Materials and Methods

### 2.1. Subjects

A total of 85 ASD preschoolers were recruited from November 2015 to February 2018 during a randomized clinical trial whose primary outcome was to evaluate the efficacy of a probiotic in reducing ASD severity, measured by the Autism Diagnostic Observation Schedule-calibrated severity score (ADOS-CSS) [15,16]. To do so, children were evaluated at baseline (T_0_) and after 6 months (T_2_) of the administration of placebo or probiotic mixture. Key details of the clinical trial, comprising inclusion/exclusion criteria, randomization procedures, and sample size calculation, have been described by Santocchi et al. [14].

The probiotic supplement was De Simone Formulation, a patented mixture already approved for use in children (manufactured by Mendes SA, Lugano, Switzerland and marketed as Vivomixx^®^ in the EU and Visbiome^®^ in the USA). Each packet contains 450 billion lyophilized bacterial cells belonging to eight probiotic strains: one strain of *Streptococcus thermophilus* NCIMB 30438, three strains of *Bifidobacterium* (*B. breve* NCIMB 30441, *B. longum* NCIMB 30435, and *B. lactis* NCIMB 30436), and four strains of *Lactobacillus* (*L. acidophilus* NCIMB 30442, *L. plantarum* NCIMB 30437, *L. paracasei* NCIMB 30439, and *L. helveticus* NCIMB 30440).

In addition to ADOS score evaluation, fecal samples were also collected, allowing complete microbiota and metabolome evaluations at both T_0_ and T_2_ on a subset of 57 of the 85 subjects originally recruited. As detailed in Table 1, 61% of the 57 children (35) were characterized by medium ASD severity, while 7% and 32% presented low and high forms of ASD, respectively. ASD was also associated with gastrointestinal symptoms (GI) in 32% and 33% of the children with medium and high levels of ASD, respectively, while no GI cases were evidenced in the Low ASD group. Overall, NGI and GI children were 40 and 17, respectively.

Table 1 also shows that the double-blind randomized assignment to the treatments with placebo or probiotic made in the original cohort of 85 children ended up in an allocation close to 1:1 in the restricted group of 57 children now investigated, both when considering and not considering the further grouping according to the severity of autism.

### 2.2. Clinical Assessments

A team of experts conducted an ASD diagnosis based on DSM-5 criteria confirmed by the Autism Diagnostic Observation Schedule, Second Edition (ADOS-2) [15]

The ADOS-calibrated severity score (ADOS-CSS) [17,18] was used to standardize and compare ADOS-2 raw scores across different modules and ages. The participants were divided into three subgroups based on their ADOS-CSS scores: low severity (1–4), moderate severity (5–7), and high severity (8–10). Validated cut-offs were used for these three categories. A modified version of the GI severity index (GSI [19]) was used to detect GI symptoms to classify the subjects as GI vs. NGI. A total score of 4 or above (with at least 3 score points from the first six items) was considered clinically significant for classifying a subject within the GI group.

### 2.3. Metabolomics Analysis by ^1^H-NMR

Metabolomics analysis followed the method by Laghi et al. [16]. First, D_2_O was used as a solvent for a solution of 3-(trimethylsilyl)-propionic-2,2,3,3-d_4_ acid sodium salt (TSP) 10 mM and NaN_3_ 2 mM. A pH of 7.00 ± 0.02 was granted by a phosphate buffer 1 M.

In an Eppendorf tube, 1 mL of deionized water was vortex mixed with 80 mg of each fecal sample, followed by centrifugation at 4 °C for 15 min at 18,630× *g*. The supernatant (0.7 mL) was mixed with 0.1 mL of the above-described solution. After a further centrifugation step, ^1^H-NMR spectra were registered.

A spectrometer AVANCE III from Bruker (Milan, Italy), set at 600.13 MHz and equipped with the software Topspin 3.6, was used to register the ^1^H-NMR spectra at 298 K. The suppression of the residual water signal was granted by presaturation, while broad signals from large molecules were reduced by a CPMG-filter, as previously described [16]. Each spectrum was acquired by summing up 256 transients, separated by a relaxation delay of 5 s, using 32 K data points spanning a 7184 Hz spectral window, leading to an acquisition time of 2.28 s.

Differences in water and solids content among samples were considered by probabilistic quotient normalization [20]. Spectrum phase was manually adjusted in Topspin, while in R computational language [21] the residual water signal was removed and the baseline of the spectrum was corrected. Signals were assigned according to Laghi et al. [16], while molecule quantification was performed by means of rectangular integration [22].

### 2.4. Microbiota Analysis

QIAamp PowerFecal DNA Kit (Qiagen, Hilden, Germany) was employed to extract the DNA from fecal samples stored at −80 °C. The samples were homogenized in a 2 mL bead beating tube with garnet beads. Solution C6 was employed to elute the DNA, which was then stored at −20 °C for subsequent real-time qPCR analysis. As reported elsewhere [23], total bacteria were quantified with a universal primer set specific for 16S rDNA of domain bacteria and conditions.

Genus-specific primers and conditions were employed to quantify lactobacilli and Bifidobacteria, as described by Stsepestova et al. [24] and Matsuki et al. [25], respectively. Primers and protocols described by Bartosch et al. [26] and Larsen et al. [27] were employed to determine the *Bacteroides*/*Prevotella* group and *Prevotella* genus, respectively. Finally, primers and PCR conditions reported by Williams et al. [28] and Collado et al. [29] were employed for the *Sutterella* genus and *Akkermansia muciniphila* species, respectively. Each sample was analyzed in duplicate and copy number values were determined by interpolating data onto standard curves. These curves were generated using serial 10-fold dilutions of DNA extracted from *Bifidobacterium breve*, *Lactobacillus brevis*, *Bacteroides fragilis*, *Prevotella pallens*, *Sutterella wadsworthensis*, and *A. muciniphila*. PCR was performed on optical-grade 96-well plates with Power SYBR GREEN PCR Master Mix (Applied Biosystems, Foster City, CA, USA) using the Applied Biosystems 7500 real-time PCR instrument.

### 2.5. Statistical Analysis

Statistical analysis has been conducted in R computational language [21], while artwork was refined by GIMP (version 2.10, www.gimp.org). Comparisons were performed by the Wilcoxon test and Chi-squared test; throughout the text, p_w_ and p_c_ denote their respective *p*-values.

Trends underlying groups of molecules were highlighted by robust principal component analysis (rPCA) models [30]. A score plot and a Pearson correlation plot summarize the main features of an rPCA model. The former represents the samples in the PC space, thus evidencing the overall structure of the data. The latter reports the correlations between each variable’s concentration and the model’s components, thus showing which molecule mostly determines the data’s structure [31]. Linear discriminant analysis was finally deployed to separate the groups based on the important metabolites arising from the rPCA model concerning probiotic treatment and GI symptoms.

## 3. Results

### 3.1. Autism Diagnostic Observation Schedule, Second Edition

The ADOS-2 assessment was performed by Santocchi et al. in 85 children [15]. The present study was conducted on a subset of 57 children, for whom complete ADOS-2, fecal metabolome, fecal microbiota, and fecal calprotectin data were available at both T_0_ and T_2_. Their main characteristics are reported in Table S1. As detailed in Table 2, 29, and 28 children pertained to the placebo and probiotics groups, respectively.

As a preliminary step, we evaluated whether the subset of 57 subjects yielded ADOS results consistent with those observed in the full cohort of 85 participants. No significant changes in the ADOS score following treatment were found when all children were analyzed as a single group, while the NGI children showed a significant difference in the response to the two treatments (p_w_ = 0.044), with a 0.19 increase of the ADOS-2 score when the placebo was administered and a 0.68 decrease when the probiotic was administered, on average. Comparing numbers, 10 NGI showed an increase of ADOS-2 scores after the treatment with placebo, compared to only 3 after the treatment with the probiotic. Conversely, 6 children showed an improvement (lower ADOS-2 scores) after the treatment with placebo, compared to 8 after the treatment with the probiotic.

No clear trend was observed in GI children treated either with placebo or probiotic. Only 3 cases in the placebo group had better ADOS-2 values, while probiotic led to an equal number of subjects with better, worse, and identical scores.

### 3.2. Fecal Metabolome ADOS-2 Related

Metabolomic analysis of NMR spectra resulted in the identification and quantification of 59 metabolites for each sample, including for instance organic acids, amino acids, alcohols, and sugars (Table S4). The variation of their concentration between T_0_ and T_2_ has been studied to highlight potential metabolic pathways affected by treatment with placebo/probiotics for children with/without (GI/NGI) gastrointestinal symptoms at baseline. In the first step, presented in this chapter, we explored the variations of ADOS-2 related metabolites by adopting the model presented by Laghi et al. [14] based on the samples collected at T_0_.

**Figure 1:**
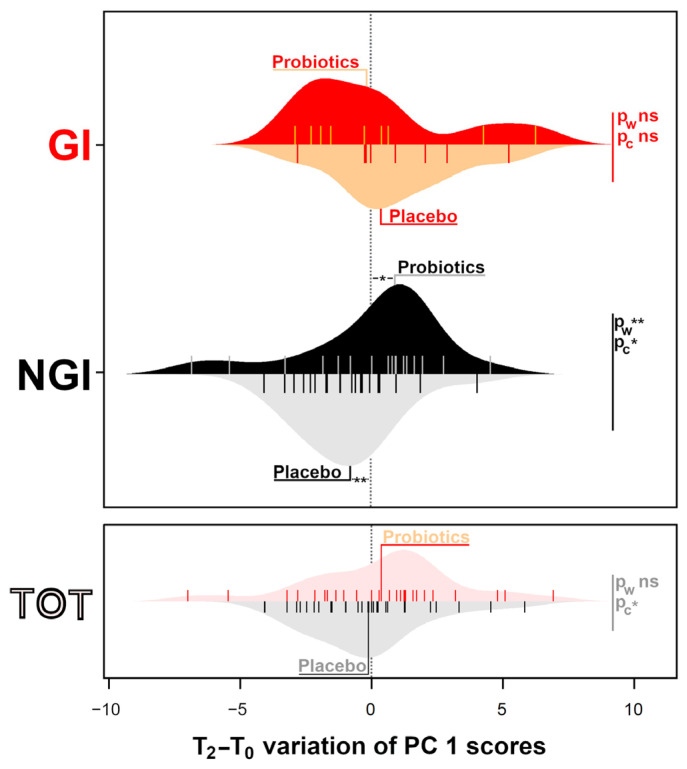
Beanplot of the difference between the PC 1 scores of the samples at T_0_ and T_2_, projected over the rPCA model outlined in Figure 1 of the paper by Laghi et al. [14] The beans report the data for all the children (TOT) or for the GI and NGI children. Each bean’s lower and upper halves report the children assigned to the Placebo and Probiotics groups, respectively. The samples are reported with vertical lines, while their distribution is reported with shapes. The wording Placebo or Probiotics are reported over the median sample. * *p* < 0.05; ** *p* < 0.01.

Laghi and colleagues set up an rPCA model where children with low, medium, and high ADOS-2 scores appeared at high, intermediate, and low PC 1 values, respectively. The consistent picture represented by this model prompted us to use it to compare the consequences of the two treatments from the point of view of the fecal metabolome. To do so, the samples obtained at T_2_ were projected over the rPCA model of Figure 1 of the paper by Laghi, the mathematical details of which are given in Table S3 of the same paper. Subsequently, the T_2_–T_0_ difference of the PC 1 scores was calculated for each child, as reported in Figure 1 and Table S2.

In Table S2, in particular, the T_2_–T_0_ difference is reported in terms of mean values and in terms of number of children whose PC 1 score increased or decreased between the two timepoints. Here, it is worth noting that the position along PC 1 is a continuous variable, so unlike ADOS-2 scores, PC 1 values do not remain unchanging. Focusing on NGI children, the probiotic had a significant impact in moving the subjects towards higher PC 1 values, associated with improved health. This was true when the PC 1 score was considered in absolute terms (*p* = 0.003) but also when the number of children with position along PC 1 decreaed or increased (*p* = 0.012). No significant differences emerged when focusing on GI children.

In summary, the psychometric evaluations (Table 1 and Table 2) and metabolomic observation of feces (Figure 1) showed that the administration of the probiotic decreased ADOS-2 scores only in children in the NGI group.

It was remarkable to notice that 6 of the NGI children had an improved ADOS-2 in connection with the administration of the placebo. We wondered whether features of the fecal metabolome of NGI children at T_0_ could help outline the metabotype of subjects with the highest chances to improve their ADOS-2 score even when receiving the placebo only. To address the issue, we compared by Wilcoxon test the levels of the molecules at T_0_ between the children whose ADOS-2 score had either increased or decreased from T_0_ to T_2_, independently of the treatment (Table 3). We identified 4 molecules, namely 4-hydroxyphenylacetate, tyramine, proline, and galactose. In detail, children expected to improve their ADOS-2 score between the two visits appeared to have, at T_0_, a significantly lower concentration of 4-hydroxyphenylacetate and tyramine, while they had a higher concentration of proline and galactose compared to the children expected to worsen their ADOS-2 score. Given the possibility of having an improvement of the ADOS-2 score, a low concentration of 4-hydroxyphenylacetate and tyramine and a high concentration of proline and galactose could be considered as desirable, as summarized in Table 3.

**Figure 2:**
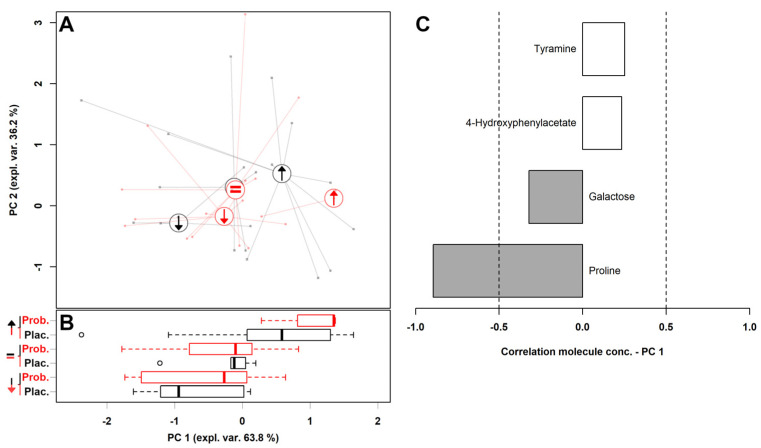
An rPCA model calculated based on the samples and molecules in Table 3. (**A**) In the scoreplot, children treated with the placebo and the probiotic are represented with black and red colors. Big circles represent the median values obtained from children set to have at T_2_ an ADOS-2 score higher than T_0_ (↑), lower than T_0_ (↓) and equal to T_0_ (=). (**B**) Boxplot summarizing the position of the groups along PC 1. (**C**) Correlation plot reporting the correlation between the importance of each substance over PC 1 and its concentration. Gray bars highlight significant correlations (*p* < 0.05).

For a confirmation that those molecules were indeed linked to the possibility of children improving at T_2_, we calculated an rPCA model on them, considering only children whose ADOS-2 score at T_2_ was either lower (14 children) or higher (13 children) than the one at T_0_ (Figure 2). Then, over such reduced space, we projected the samples from children who showed an ADOS-2 score at T_2_ equal to that at T_0_. The first PC of the rPCA model summarized the destiny of the ADOS-2 score, with children set to have a lower and a higher score appearing at negative and positive PC 1 scores, respectively. Children set to be characterized by an equal ADOS-2 score indeed appeared at intermediate PC 1 scores. To verify this visual impression, we compared the distances between samples from children with equal ADOS-2 scores to those with lower and higher ADOS-2 scores. We found that the distance between samples from children with lower and higher ADOS-2 scores was significantly greater than the other comparisons (*p* < 0.001).

In addition, the children set to improve their ADOS-2 score between the two visits in connection to the treatment with the placebo (represented with a black arrow pointing down), appeared at significantly (*p* < 0.01) lower PC 1 scores than those improving in connection to the probiotic administration (represented with a red arrow pointing down). In parallel, the children set to worsen their ADOS score between the two visits in connection to the treatment with the placebo (represented with a black arrow pointing up), appeared at significantly (*p* < 0.01) lower PC 1 scores than those improving in connection to the probiotic administration (represented with a red arrow pointing up).

### 3.3. Fecal Microbiota and Calprotectin

In the feces of the 57 children that we could observe at both T_0_ and T_2,_ we quantified the relative abundance of lactobacilli, bifidobacteria, *Akkermansia muciniphila*, *Bacteroides*, *Prevotella*, and *Sutterella*, as detailed in Table S3. Two-tailed Wilcoxon tests were applied on the difference T_2_-T_0_ in the relative abundance of each microorganism species and resulted in no significant difference in bifidobacteria, *Akkermansia muciniphila*, *Bacteroides*, and *Prevotella* between children treated with probiotics or placebo. The same test showed, on the contrary, that the relative abundance of lactobacilli and *Sutterella* changed significantly due to the administration of probiotics between the two visits.

**Figure 3:**
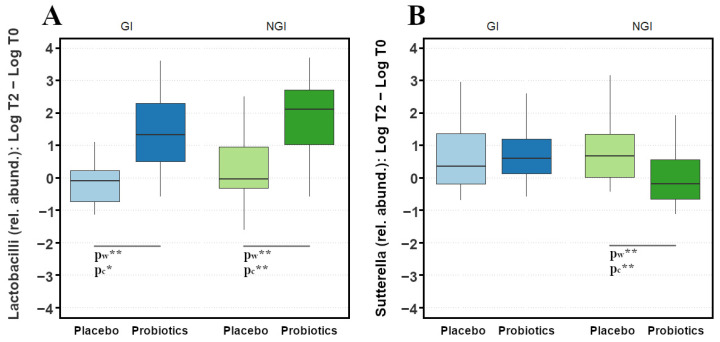
Boxplot of the difference Log_10_ T_2_–T_0_ in the relative abundance of (**A**) lactobacilli, (**B**) *Sutterella*. Significance of the differences are expressed as p_w_ and p_c_ for *p* < 0.1 (*) and *p* < 0.05 (**) between Placebo and Probiotics groups according to Wilcoxon and Chi tests, respectively.

As evidenced in Figure 3A, probiotics and placebo led to a significantly different trend in lactobacilli abundance, both for GI (p_w_ = 0.032) and NGI children (p_w_ = 0.002), where probiotics increased the relative abundance of lactobacilli of 1.32 (1.79) and 2.10 (1.69) Log_10_ median values (IQR), respectively, while the Placebo group remained unchanged. By expressing the differences in terms of number of children with increasing values, a Chi test confirmed the results from Wilcoxon test for NGI children (p_c_ = 0.018) and, although with a lower confidence level, for GI subjects (p_c_ = 0.088).

Figure 3B displays data related to *Sutterella* relative abundance. Generally, higher values were reported after 6 months for all the groups except for NGI subjects treated with probiotics. Indeed, a decrease in the relative abundance was evidenced and resulted in a significant difference when placebo and probiotics were administered to children, considering two-tailed Wilcoxon (p_w_ = 0.035) and Chi tests (p_c_ = 0.028).

Intestinal inflammation in each child at the two timepoints was evaluated by fecal calprotectin. Its concentration was subjected to the same reasoning and calculations described above for fecal microbiota. No significant differences emerged in any case.

In parallel to what we did for the fecal metabolome, we wanted to assess whether the concentrations of some microorganisms at T_0_ could determine the variations of ADOS-2 between T_0_ and T_2_. To address the issue, first, we compared, by Wilcoxon test, the abundance of the microorganisms at T_0_ between the children whose ADOS-2 score had either increased (13) or decreased (14) from T_0_ to T_2_, independently of the treatment. Then, we repeated the same test on the children whose ADOS increased (10) or decreased (6) when treated only with placebo. In no case did significant differences emerge.

### 3.4. Fecal Metabolome Microbiota Related

The second step of the metabolome analysis, presented in this chapter, is prompted by the results achieved in the preceding sections, which illustrated that the probiotic and placebo treatments influenced the relative abundance of lactobacilli and *Sutterella* differently. Consequently, we expected that the treatment could affect the fecal metabolome differently. To further investigate the changes in the metabolome, the concentration of the molecule at T_0_ was subtracted from those at T_2_, similarly to the approach used for microbiota data. Two parallel exploratory approaches were used on those delta-time data to find which of the 59 metabolites changes significantly over time.

As a first approach, the Wilcoxon test was applied to each metabolite to analyze the different concentrations by grouping: NGI children treated with probiotics vs. placebo and GI children treated with probiotics vs. placebo. Due to the exploratory target of the analysis, we set the *p*-value at 0.1. Thus, only the metabolites that showed a significant value lower than the set target, for at least one group (NGI or GI), were considered important. The outcome of the analysis is presented in Table 4. The analysis allowed us to find 9 important metabolites, which significantly varied between placebo and probiotics, considering the group of children without gastrointestinal symptoms (NGI). On the contrary, none of the metabolites were significantly affected by treatment in GI subjects. It is worth noting that the GI group often showed higher variability, expressed in IQR, which could be an effect of the gastrointestinal symptoms, and resulted in a lack of consistent variations between placebo and probiotics.

**Figure 4:**
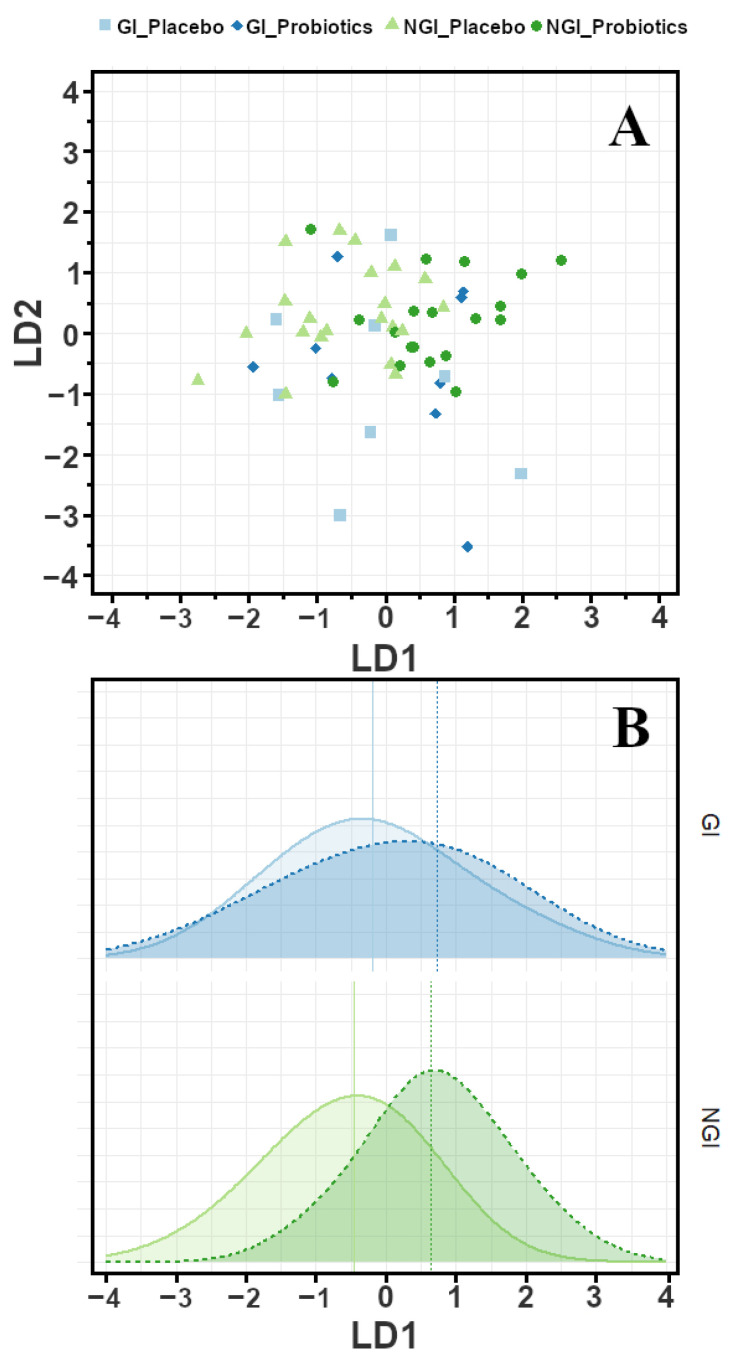
LDA model based on the concentration (T_2_-T_0_) of 5 metabolites: acetate, propionate, butyrate, isobutyrate, and tryptophan for GI and NGI children. Score plot (**A**) and distribution curves (**B**) of LD1 scores for Placebo (solid line) and Probiotics (dashed line), while vertical segments are the corresponding median values.

A second analysis of delta-time data was carried out in parallel to the Wilcoxon test. We created a new unsupervised rPCA model to explore the variance of all metabolites between the two visits. In that new model, PC1 and PC2 described, respectively, 71.5% and 11.0% of the total variance. Interestingly, the four major contributors to PC1, namely acetate, propionate, butyrate, and isobutyrate, were already reported in Table 4 as a result of the statistical test. As a last step of data investigation, we implemented a linear discriminant analysis (LDA) to cluster the four groups, NGI/GI treated with placebo/probiotics, based on the important metabolites. The best discriminant ability was achieved by building the model based on the concentration change of five metabolites. Those are the abovementioned molecules: acetate, propionate, butyrate, and isobutyrate, plus tryptophan, which was found to be the most significant metabolite by means of Wilcoxon test. The ability of LDA to describe the different groups is illustrated in Figure 4 where LD1 plays the greatest role as a discriminator of both GI and NGI. It is worth noticing that the median of each distribution curve is placed at similar values on LD1 for each treatment and regardless of the gastrointestinal symptoms, namely −0.186/−0.455 (GI/NGI) for Placebo and 0.731/0.642 (GI/NGI) for Probiotics. In terms of overall classification performance of the LDA model, only NGI subjects were correctly separated and assigned to the Placebo and Probiotics groups with an 82.5% correct classification rate, while a poor discrimination rate was achieved for GI (13.3%). This aligns with the quantitative results of Table 4, confirming that the metabolites significantly changed only in children without gastrointestinal symptoms.

## 4. Discussion

This study aims to identify markers in the water-soluble fecal microbiota and metabolome and their link to the administration of probiotics in ASD preschoolers. Starting from data presented by Santocchi et al. [15] and Laghi et al. [16], this work includes the evaluation of the microbiota and metabolome of samples collected at the beginning and after six months of RCT intervention. The analysis of the metabolome was split into two parts: the first part of the investigation aimed at finding metabolites that change over time as a function of modifications of the ADOS-2 scores, while the second part pointed out the metabolites correlated to the microbiota as a function of DSF probiotic treatment. Santocchi et al. highlighted that ASD children with and without GI symptoms could represent two different populations, and probiotic interventions could potentially provide different effects, because of distinct microbiota targets. Therefore, the analysis classifies the microbiota and metabolome of the 57 children enrolled in 4 groups: NGI/GI symptoms treated with probiotics/placebo.

### 4.1. Change in the Metabolome Related to ADOS-2 Score

After 6 months of intervention, a total of 20 subjects reported a better (lower) ADOS-2 score. Those were equally distributed in GI children, i.e., 3 children treated with probiotics and placebo, respectively, while, in NGI subjects, 8 improved with probiotics and 6 others with placebo only. The children set to improve the ADOS-2 score in connection with the placebo appeared at a lower PC1 score than those receiving the probiotics, a condition that could be informally described as more favorable. This allows us to deduce that children set to improve in connection with the probiotic started from a less favorable condition than those improving due to the placebo.

An in-depth analysis of the initial conditions showed that some metabolites were recurrently connected with reducing ASD symptoms after the observation period, independently of the received treatments. Among them, we found a generically low concentration of 4-hydroxyphenylacetate and tyramine in children who later improved their conditions. Those metabolites are derived from the metabolism of aromatic amino acids and are potentially produced by gut microbiota in altered gastrointestinal conditions. Similarly to our achievement, Kang and colleagues [32] found that those molecules were different in the fecal metabolome of children with ASD compared to typically developing controls. Moreover, after their treatment through microbiota transfer therapy, the level of those metabolites did not significantly change, confirming that their concentration could only be ascribed to the microbiota composition typical of ASD conditions, and hardly modifiable by interventions.

As mentioned in Table 3, their initial low concentrations are an index of a potential improvement—treatment-independent—of ADOS-2 score after 6 months. Similarly, we found that high initial concentrations of proline and galactose can lead to better conditions after the same period. Proline, an amino acid, and galactose, a carbohydrate, can be metabolized by several gut microorganisms and lead to a generic improvement of gut functionality, including anti-inflammatory and barrier functions [33]. Indeed, the high level of galactose at gut level is the goal of galacto-oligosaccharide-based prebiotics, which aim at balancing microbiota diversity [34]. Proline, likewise, can modulate the structure and composition of microbiota, and its supplementation in mice has been reported to induce benefits on autism-like behaviors in that studied model [35].

It is worth underlining that the four metabolites described in the present chapter are specific characteristics of the metabolome in ASD children who improved their behavior over time, independently of our placebo or probiotic treatment. Notwithstanding, the mechanism of action is only partially elucidated in literature; they can represent alternative supplementation targets.

### 4.2. Effects of Probiotics Administration on Fecal Microbiota and Metabolome

The administration of probiotics to ASD preschoolers showed a general change in the microbiota balance over the six months of analysis. The fecal microbiota observed at T_2_ was influenced by the higher abundance of lactobacilli when probiotics were administered to children with or without gastrointestinal disease. The lactobacilli population’s significant increase in DSF probiotics intake has been reported to have multiple beneficial impacts on the microbiota. For instance, the competition against pathogenic microorganisms by impeding their proliferation or their adhesion to epithelial cells, the improvement of gastrointestinal barrier function, and the support in the maintenance of homeostasis in intestinal disorders, which protects against inflammatory diseases, are desirable consequences of probiotics described in the literature [36,37,38]. As evidenced by Laghi, their abundance is negatively correlated with molecules that characterize high-ADOS-2 scores. That evidence is confirmed in this study, which considers the same children after six months of the DSF supplement.

Among the different species analyzed in this work, we noticed a significant reduction in the relative abundance of *Sutterella* when DSF probiotics were supplemented to NGI subjects. After six months, the potential competition of species induced by probiotics might have reduced the proliferation of *Sutterella*. This aspect might be a key beneficial effect of probiotics since *Sutterella* has been reported as causing infections under certain conditions [28], promoting proteolytic functions and potentially involved in gut inflammation and disease processes [39]. Conversely, a lower amount of *Sutterella* was found in ASD children with functional gastrointestinal disorders than their HC peers [40]. In the current study, we observed that NGI children who improved their conditions between the timepoints showed, on average, lower *Sutterella* abundance along with a lower ADOS score.

Along with fecal microbiota, the fecal metabolome showed differences between the two time points (T_0_ and T_2_), which are mainly ascribed to two classes of metabolites: short-chain fatty acids (SCFAs) and free amino acids (FAAs).

In our study, statistical significance, even if not corrected for multiple comparisons, was observed for the four key SCFA compounds, which principally accounted for the explanation of the variance of delta-time data (T_2_-T_0_). Acetate, propionate, butyrate, and isobutyrate are, indeed, the most quantitatively significant short-chain fatty acids produced from fermentation in the gut (accounting for 90–95%), and they are known to play a positive role in the balance of the microbiota [41]. Several studies have reported that SCFA formation, mainly from dietary fiber, has a beneficial anti-inflammatory effect, regulates pH, contributes to the host homeostasis, protects the gut barrier, and prevents several related diseases [36,42]. In this context, probiotic administration and a fiber-rich diet have a recognized positive effect on SCFA levels, and their formation pathways are described in the literature [42,43]. In particular, the abundance of Lactobacilli is mainly associated with a relevant production of lactic acid and, for certain species, of acetate, while propionate and butyrate are commonly the end results of fermentation of other species [44]. *Sutterella*, on the other hand, has not been reported to produce significant amounts of SCFAs. Previous investigations detected altered fecal levels of SCFA in ASD patients, although some conflicting results emerged in the scientific literature. Indeed, both lower [8], elevated [45], or no statistically significant differences [46,47] between individuals with ASD and typically developing peers were detected in fecal levels of SCFA.

This research found a negative trend between the total SCFA content in feces and the relative abundance of lactobacilli. Considering only NGI subjects, lactobacilli showed higher abundance in the group treated with probiotics, and *Sutterella*, conversely, was lower. In parallel, the content of acetate, proprionate, butyrate, and isobutyrate was lower after six months when the probiotic group was compared to the placebo group. This indicates that the intake of lactobacilli through the probiotic administration does not promote a positive effect through the production of SCFAs, but the potential beneficial effects of probiotics should be attributed to other mechanisms of action. In any case, SCFA changes over time indicate a different balance in the microbiota, potentially due to the competition for the ecological niche, which can be exploited as a marker of the probiotic action.

A second group of metabolites, which arose from the analysis of the fecal metabolome T_2_–T_0_, are free amino acids (FAA). Specifically, three essential amino acids, tryptophan, valine, and leucine plus aspartate. The alteration of amino acid composition, i.e., higher amounts of valine, leucine, and aspartate linked with a different balance of the microbiota, was not unexpected. Indeed, researchers have underlined that gut microbiota species such as *Bacteroides*, *Clostridium*, *Bifidobacterium*, and *Lactobacillus* are involved in proteolytic reactions which may lead to the release of FAA [14,48]. Among the other FAA, the change of aspartate concentration has often recurred in studies that deal with ASD. Its action on *N*-methyl-D-aspartate (NMDA) receptors might cause its dysfunction, which, in turn, can be associated with ASD symptoms. Emerging evidence indicates that d-aspartate and d-serine play key roles as neuromodulators in glutamatergic transmission [49]. These findings suggest that targeting NMDA receptors could offer promising therapeutic potential for ASD, and experimental studies are already investigating this approach [50].

Tryptophan is the only amino acid that showed opposite trends as a function of probiotic/placebo treatments. We found a lower concentration after six months in NGI children treated with DSF probiotics, which was positively correlated with a lower concentration of nicotinate. Nicotinate is the chemically active form of niacin (vitamin B3), and it is involved as an intermediate product in the kynurenine pathway. The kynurenine pathway is the process of metabolizing tryptophan and transforming it into kynurenic and picolinic acid, kynuramines, NAD^+^, and ATP; it is thus linked to cellular energy metabolism [51]. Low amounts of both tryptophan and nicotinate might indicate an under-regulation of that pathway in the NGI subject treated with probiotics. In fact, probiotics based on lactobacilli strains, have demonstrated a potential influence on host tryptophan metabolism, according to O’Mahony [52]. The consequences of this are debated in the scientific literature. Imbalances in the kynurenine pathway could lead to low NAD+ levels and, consequently, to mitochondrial dysfunction, which potentially contributes to cognitive and neuropsychiatric disorders [53]. The alteration of the same metabolic pathway could also modify the quinolinic–kynurenic acid balance, accountable for possible ASD related issues [54]. Furthermore, the same study reported that external supplements of tryptophan, aiming at balancing the related pathways, could be an effective tool. However, the treatment’s effectiveness on the host’s tryptophan level is still unclear. The imbalance of tryptophan metabolism is also a key effect of the microbial influence on the gut–brain axis, thus, on serotonin production, which is deeply associated with mood and cognition [52], frequently connected to ASD symptoms [55].

In our study, we could not quantitatively correlate the concentration of SCFAs, either as individual metabolites or their sum, or the content of amino acids with ADOS-2 score. Nevertheless, it is interesting to notice common trends that promote some compounds as interesting biomarkers for the study of ASD and the effects of probiotics administration.

In this respect, acetate, aspartate, and leucine were also part of the model developed by Laghi et al. [16], which correlates those metabolites with children with low/high ADOS-2 and with/without gastrointestinal disease. Acetate was lower, on average, in subjects with low ADOS-2, and lower in children without gastrointestinal disease (NGI) at T_0_. Similarly, in the present work, we found that, after six months from treatment (T_2_), the amount of acetate decreased when children were treated with probiotic compared to placebo. Even if the data do not allow us to establish specific quantitative thresholds, the recurrent presence of this metabolite across various analyses supports its role as a biomarker. Specifically, its low concentration seems advantageous in various respects.

Using the same approach, we noticed that aspartate changed its concentration in opposite directions according to probiotics (higher) and placebo (lower) when the same subjects were observed at T_0_ and T_2_. Interestingly, Laghi also showed a negative correlation between *Sutterella* and aspartate, which was confirmed in NGI children observed, in the present work, at a time difference of six months. Another aspect reported in the previous research is that a high concentration of aspartate is correlated with low-ADOS-2 scores, regardless of the gastrointestinal symptoms. Similar results and conclusions regarding aspartate could be applied to leucine concentrations. Indeed, a negative link between *Sutterella* and leucine was found, and subjects with a low ADOS-2 score presented higher concentration of these amino acids [16]. Although the underlying mechanism remains controversial in the literature, an increase in aspartate and leucine levels in both studies appears to be an indicator of improved conditions and DSF probiotics administration can play a positive role.

## 5. Conclusions

The present work describes an ancillary study of a randomized clinical trial conducted to observe whether a probiotic mixture could reduce the severity of ASD in preschool children, some of which are affected by gastrointestinal symptoms. Despite its secondary nature and the small sample size, with some groups particularly underrepresented, this work suggests that fecal metabolomics may be useful for monitoring and potentially for predicting changes in ASD severity following probiotic administration, while also providing insights into the underlying biological mechanisms driving these changes.

## Figures and Tables

**Table 1:** Partition of the subjects according to ASD severity, presence of GI symptoms, and treatment administration.

ADOS	Low 4		Medium 35		High 18		TOT
NGI/GI	NGI 4	GI 0	NGI 24		GI 11	NGI 12	GI 6
Placebo	3	-	11	5	7	3	29
Probiotic	1		13	6	5	3	28

**Table 2:** For the subset of 57 children, differences T_2_–T_0_ in ADOS-2 score, in terms of absolute value and in terms of number of children.

	Gastrointestinal Disease at T_0_					
	NGI			GI		
	Placebo (TOT = 21)	Probiotic (TOT = 19)	*p*	Placebo (TOT = 8)	Probiotic (TOT = 9)	*p*
ADOS-2 T_2_-T_0_	+0.19 (±1.86)	−0.68 (±1.49)	0.044 *	−0.5 (±0.76)	−0.22 (±1.56)	0.51 *
Subj. with ADOS-2 = ^†^	5	8	0.10 **	5	3	0.18 **
Subj. with ADOS-2 ↑	10	3		0	3	
Subj. with ADOS-2 ↓	6	8		3	3	

* Wilcox one-tailed test; ** Chi test; ^†^ ADOS-2 unchanged (=), increased/worsened (↑) or decreased/improved (↓).

**Table 3:** Concentration (mmol/L; median (IQR)) of the molecules in feces collected at T_0_ from children whose ADOS-2 score was set to decrease or increase at T_2_, independently of the treatment in the NGI group.

	ADOS-2 at T_2_ Lower Than T_0_ (n = 14)		ADOS-2 at T_2_ Higher Than T_0_ (n = 13)		T_0_ Levels in Subjects with Improved ADOS-2 at T_2_	*p*-Value Lower vs. Higher ADOS-2
	Placebo	Probiotic	Placebo	Probiotic		
4-Hydroxyphenyl acetate	8.54 × 10^−5^ (4.34 × 10^−5^)	8.57 × 10^−5^ (3.51 × 10^−5^)	1.28 × 10^−4^ (1.42 × 10^−4^)	1.34 × 10^−4^ (1.13 × 10^−5^)	Low	0.017
Proline	9.48 × 10^−4^ (2.17 × 10^−4^)	8.25 × 10^−4^ (2.68 × 10^−4^)	6.42 × 10^−4^ (5.42 × 10^−4^)	3.48 × 10^−4^ (1.47 × 10^−4^)	High	0.032
Galactose	1.78 × 10^−4^ (1.93 × 10^−4^)	1.80 × 10^−4^ (2.62 × 10^−4^)	1.23 × 10^−4^ (4.48 × 10^−5^)	1.11 × 10^−4^ (8.29 × 10^−6^)	High	0.041
Tyramine	3.73 × 10^−5^ (2.01 × 10^−5^)	5.93 × 10^−5^ (5.36 × 10^−5^)	8.88 × 10^−5^ (6.80 × 10^−5^)	1.13 × 10^−4^ (2.79 × 10^−5^)	Low	0.046

**Table 4:** Concentration (mmol/L; median (IQR)) of the molecules in feces expressed as difference T_2_-T_0_ with significant *p*-value (Wilcoxon test) between placebo (control) and probiotic treatment.

	Gastrointestinal Disease at T_0_					
	NGI			GI		
	Placebo (TOT = 21)	Probiotics (TOT = 19)	*p*	Placebo (TOT = 8)	Probiotics (TOT = 9)	*p*
Acetate	9.29 × 10^−3^ (2.65 × 10^−2^)	1.68 × 10^−3^ (2.68 × 10^−2^)	0.031	2.54 × 10^−4^ (2.82 × 10^−2^)	−2.42 × 10^−2^ (7.10 × 10^−2^)	n.s
Aspartate	−2.05 × 10^−4^ (7.77 × 10^−4^)	1.02 × 10^−4^ (7.02 × 10^−4^)	0.014	1.87 × 10^−4^ (8.14 × 10^−4^)	1.29 × 10^−4^ (1.12 × 10^−3^)	n.s
Butyrate	1.94 × 10^−3^ (7.64 × 10^−3^)	−1.84 × 10^−3^ (9.57 × 10^−3^)	0.078	2.67 × 10^−4^ (1.18 × 10^−2^)	−8.25 × 10^−3^ (3.01 × 10^−2^)	n.s
Isobutyrate	7.08 × 10^−4^ (4.02 × 10^−3^)	−1.18 × 10^−3^ (2.44 × 10^−3^)	0.008	−2.51 × 10^−5^ (4.60 × 10^−3^)	−3.63 × 10^−3^ (6.01 × 10^−3^)	n.s
Leucine	−2.46 × 10^−4^ (8.33 × 10^−4^)	4.42 × 10^−4^ (1.69 × 10^−3^)	0.041	3.56 × 10^−4^ (1.77 × 10^−3^)	−5.03 × 10^−4^ (1.32 × 10^−3^)	n.s
Nicotinate	3.75 × 10^−5^ (9.20 × 10^−5^)	−2.50 × 10^−5^ (1.22 × 10^−4^)	0.031	−4.79 × 10^−5^ (7.36 × 10^−5^)	4.24 × 10^−6^ (8.91 × 10^−5^)	n.s
Propionate	4.65 × 10^−3^ (1.31 × 10^−2^)	−4.35 × 10^−3^ (7.27 × 10^−3^)	0.007	−4.64 10^−4^ (1.73 10^−2^)	−1.05 × 10^−2^ (2.11 × 10^−2^)	n.s
Tryptophan	2.34 × 10^−5^ (6.57 × 10^−5^)	−5.46 × 10^−5^ (8.10 × 10^−5^)	<0.001	−1.67 × 10^−5^ (1.28 × 10^−4^)	7.43 × 10^−6^ (5.34 × 10^−5^)	n.s
Valine	−2.41 × 10^−4^ (5.97 × 10^−4^)	2.27 × 10^−4^ (8.88 × 10^−4^)	0.061	4.49 × 10^−5^ (8.66 × 10^−4^)	−2.95 × 10^−4^ (4.55 × 10^−4^)	n.s

## Data Availability

The original contributions presented in this study are included in the article/supplementary material. Further inquiries can be directed to the corresponding author.

## Supplementary Materials

The following supporting information can be downloaded at: https://www.mdpi.com/article/10.3390/metabo16040262/s1, Table S1: Characteristics of the subjects in the whole sample and divided by autism severity; Table S2: Differences in PC 1 value between the visits T0 and T2 connected to the treatment, in terms of absolute value and in terms of number of children with ADOS improved or worsened; Table S3: For the 57 children with complete metabolomics and microbiota data, difference between total bacteria and each microorganism evaluated, at T0 and at T2, expressed as median (±MAD). Children are separated in NGI, GI, and overall considered; Table S4: In the feces of the 57 children with complete metabolomics and microbiota data, concentration of molecules at T0 and at T2, expressed as median (±MAD).

## Abbreviations

The following abbreviations are used in this manuscript:

ASD	Autism Spectrum Disorder
ADOS	Total Autism Diagnostic Observation Schedule—Calibrated Severity Score
GI	Group with gastroinestinal symptoms
NGI	Group without gastroinestinal symptoms
^1^H-NMR	Proton Nuclear Magnetic Resonance Spectroscopy
rPCA	Robust Principal Components Analysis
