# Supplementary material for: Effects of Probiotic Supplementation on Gut Microbiota and Fecal Metabolome in Autism Spectrum Disorders: A Secondary Analysis of a Randomized Clinical Trial in Preschoolers
Source: Metabolites. 2026 Apr 13;16(4):262. doi: 10.3390/metabo16040262 (PMC13117773; doi:10.3390/metabo16040262)
Supplement: Supplementary file 1 [file metabolites-16-00262-s001.zip › metabolites-4226374-supplementary.pdf]

Effects of Probiotic Supplementation on Gut Microbiota and Fecal Metabolome in Autism Spectrum Disorders: A Secondary Analysis of a Randomized Clinical Trial in Preschoolers

Table S1. Characteristics of the subjects in the whole sample and divided by autism severity

	Whole-Sample	Low-ADOS	Moderate-ADOS	High-ADOS
n	57	4	35	18
NGI/GI	40/17	4/0	24/11	12/6
Females/Males	12/45	1/3	8/27	3/15
Age (years)	4.19 ± 1.14	3.83 ± 1.20	4.43 ± 1.14	3.79 ± 1.05

Abbreviations: NGI, Children without gastrointestinal symptoms; GI, Children with gastrointestinal symptoms.

Table S2: Differences in PC 1 value between the visits T_0 and T_2 connected to the treatment, in terms of absolute value and in terms of number of children with ADOS improved or worsened.

	Gastrointestinal disease at T_0						Overall		
	NGI			GI					
	Placebo	Probiotic	*p*	Placebo	Probiotic	*p*	Placebo	Probiotic	*p*
T2-T0	-0.86 (±1.84) *p=0.003*	0.02 (±2.77) *p= 0.044*	*0.002*	+0.99 (±3.10) *p=0.54*	+0.3 (±2.43) *p=0.46*	*0.19*	-0.35	+0.11	*0.054*
PC 1 scores ↑	5	13	0.012	4	4	1	9	17	0.047
PC 1 scores ↓	16	6		4	5		20	11	

Table S3: For the 57 children with complete metabolomics and microbiota data, difference between total bacteria and each microorganism evaluated, at T_0 and at T_2, expressed as median (±MAD). Children are separated in NGI, GI and overall considered.

T_0	NGI		GI		Overall	
	Placebo	Probiotic	Placebo	Probiotic	Placebo	Probiotic
TOT bacteria - Lactobacilli	5.07 (±0.68)	5.53 (±0.66)	5.08 (±1.32)	5.25 (±1.3)	5.07 (±0.79)	5.47 (±0.74)
TOT bacteria - Akkermansia	2.65 (±1.06)	3.53 (±2.23)	6.69 (±0.68)	3.51 (±1.81)	3.69 (±2.4)	3.52 (±2.06)
TOT bacteria - Bifidobacteria	1.68 (±0.37)	1.69 (±0.26)	1.89 (±0.55)	1.79 (±0.25)	1.74 (±0.29)	1.71 (±0.24)
TOT bacteria - Bacteroides	1.23 (±0.38)	1.17 (±0.41)	0.92 (±0.28)	1.13 (±0.36)	1.22 (±0.38)	1.15 (±0.39)
TOT bacteria - Prevotella	5.97 (±0.8)	6 (±0.6)	6.53 (±1.18)	6.23 (±0.45)	5.98 (±0.9)	6.12 (±0.69)
TOT bacteria - Sutterella	3.83 (±0.85)	3.38 (±0.77)	3.33 (±0.68)	3.58 (±0.91)	3.74 (±0.78)	3.47 (±0.82)

T_2	NGI		GI		Overall	
	Placebo	Probiotic	Placebo	Probiotic	Placebo	Probiotic

TOT bacteria - Lactobacilli	4.57 (±1.07)	3.42 (±0.43)	5.14 (±1.05)	3.91 (±0.9)	4.7 (±1.22)	3.46 (±0.62)
TOT bacteria - Akkermansia	3.05 (±1.65)	4.17 (±3.26)	4.02 (±2.11)	3.17 (±1.42)	3.6 (±2.12)	3.65 (±2.21)
TOT bacteria - Bifidobacteria	1.82 (±0.42)	1.82 (±0.36)	1.96 (±0.54)	1.91 (±0.28)	1.86 (±0.47)	1.84 (±0.31)
TOT bacteria - Bacteroides	0.96 (±0.27)	1.14 (±0.43)	0.93 (±0.23)	0.99 (±0.18)	0.96 (±0.27)	1.07 (±0.33)
TOT bacteria - Prevotella	5.98 (±0.74)	6.29 (±0.83)	5.94 (±0.72)	6.11 (±1.02)	5.96 (±0.77)	6.29 (±0.82)
TOT bacteria - Sutterella	3.15 (±0.47)	3.29 (±0.61)	2.55 (±0.53)	3.26 (±0.71)	3.12 (±0.83)	3.26 (±0.58)

Table S4: In the feces of the 57 children with complete metabolomics and microbiota data, concentration of molecules at T_0 and at T_2, expressed as median (±MAD).

	NGI		GI	
T0	Placebo	Probiotic	Placebo	Probiotic
Nicotinate	1.31E-04 (±3.91E-05)	1.67E-04 (±4.93E-05)	1.89E-04 (±5.50E-05)	2.36E-04 (±1.16E-05)
Formate	1.28E-04 (±2.65E-05)	9.86E-05 (±3.01E-05)	1.57E-04 (±5.37E-05)	1.69E-04 (±3.56E-05)
Hypoxanthine	1.83E-04 (±5.87E-05)	2.06E-04 (±7.12E-05)	2.23E-04 (±4.92E-05)	3.04E-04 (±9.25E-05)
Xanthine	2.30E-04 (±5.60E-05)	2.86E-04 (±1.03E-04)	3.04E-04 (±2.57E-05)	3.07E-04 (±6.80E-05)
Tryptophan	2.64E-04 (±3.16E-05)	3.16E-04 (±5.30E-05)	3.42E-04 (±3.75E-05)	2.72E-04 (±4.01E-05)
Phenylalanine	1.38E-03 (±1.46E-04)	1.39E-03 (±3.10E-04)	1.19E-03 (±1.11E-04)	1.17E-03 (±2.08E-04)
Phenylacetate	1.02E-03 (±3.45E-04)	1.00E-03 (±4.66E-04)	1.05E-03 (±4.41E-04)	8.38E-04 (±3.55E-04)
3-Hydroxyphenylacetate	1.68E-04 (±6.75E-05)	2.14E-04 (±9.68E-05)	2.69E-04 (±1.30E-04)	2.65E-04 (±5.42E-05)
Tyramine	5.14E-05 (±3.37E-05)	7.07E-05 (±4.35E-05)	1.12E-04 (±1.56E-05)	9.18E-05 (±4.66E-05)
Tyrosine	2.92E-03 (±2.80E-04)	2.97E-03 (±4.92E-04)	2.57E-03 (±3.01E-04)	2.28E-03 (±5.37E-04)
4-Hydroxyphenylacetate	1.06E-04 (±3.43E-05)	1.16E-04 (±3.99E-05)	1.24E-04 (±2.30E-05)	1.14E-04 (±4.76E-05)
pCresol	4.35E-05 (±2.84E-05)	5.54E-05 (±3.63E-05)	1.28E-04 (±6.95E-05)	5.28E-05 (±4.00E-05)
Fumarate	3.60E-04 (±1.27E-04)	3.70E-04 (±2.61E-04)	2.93E-04 (±1.60E-04)	4.99E-04 (±2.34E-04)
Orotate	4.34E-05 (±1.23E-05)	6.04E-05 (±1.45E-05)	9.07E-05 (±2.67E-05)	7.25E-05 (±2.22E-05)
Uridine	4.18E-05 (±1.78E-05)	4.54E-05 (±1.86E-05)	9.34E-05 (±2.38E-05)	6.50E-05 (±2.14E-05)
Uracil	7.21E-04 (±9.46E-05)	9.50E-04 (±3.10E-04)	6.49E-04 (±1.50E-04)	8.11E-04 (±9.71E-05)
Galactose	1.35E-04 (±3.96E-05)	1.38E-04 (±4.37E-05)	1.18E-04 (±3.56E-05)	1.90E-04 (±4.76E-05)
Xylose	1.38E-04 (±8.47E-05)	9.38E-05 (±3.97E-05)	9.41E-05 (±3.13E-05)	1.97E-04 (±1.09E-04)
Fucose	8.72E-05 (±2.90E-05)	1.23E-04 (±6.38E-05)	1.06E-04 (±3.00E-05)	1.49E-04 (±3.35E-05)
Arabinose	2.97E-04 (±1.18E-04)	2.27E-04 (±6.85E-05)	2.49E-04 (±9.80E-05)	4.44E-04 (±1.14E-04)
1.3-Dihydroxyacetone	1.19E-04 (±5.79E-05)	1.34E-04 (±6.19E-05)	1.76E-04 (±1.03E-04)	2.59E-04 (±1.29E-04)
Threonine	2.01E-03 (±3.31E-04)	2.27E-03 (±4.73E-04)	2.01E-03 (±2.51E-04)	2.00E-03 (±4.26E-04)
Serine	1.49E-03 (±3.88E-04)	1.58E-03 (±3.83E-04)	1.27E-03 (±7.28E-05)	1.36E-03 (±2.95E-04)
Glycerol	2.49E-04 (±8.83E-05)	2.63E-04 (±1.06E-04)	6.42E-03 (±6.10E-03)	7.23E-04 (±5.70E-04)
Glycine	1.97E-03 (±4.02E-04)	1.89E-03 (±5.49E-04)	1.57E-03 (±4.73E-04)	1.86E-03 (±3.75E-04)
Methanol	2.77E-04 (±8.74E-05)	1.85E-04 (±4.18E-05)	3.34E-04 (±1.76E-04)	2.94E-04 (±1.61E-04)
Proline	7.42E-04 (±2.55E-04)	7.65E-04 (±1.33E-04)	6.57E-04 (±1.53E-04)	5.82E-04 (±1.50E-04)
Glucose	4.08E-03 (±1.57E-03)	4.07E-03 (±2.04E-03)	3.08E-03 (±7.48E-04)	4.30E-03 (±2.14E-03)
Malonate	2.63E-03 (±1.27E-03)	2.52E-03 (±1.19E-03)	2.37E-03 (±1.27E-03)	2.58E-03 (±1.80E-03)
Creatine	1.05E-04 (±2.42E-05)	1.21E-04 (±2.03E-05)	1.03E-04 (±7.11E-06)	9.32E-05 (±2.10E-05)
N.N-Dimethylglycine	3.72E-05 (±1.54E-05)	5.30E-05 (±2.21E-05)	4.38E-05 (±1.20E-05)	3.38E-05 (±1.95E-05)
N-Methylhydantoin	3.04E-05 (±1.30E-05)	3.33E-05 (±1.42E-05)	2.96E-05 (±1.35E-05)	4.50E-05 (±7.13E-06)
TMA	7.83E-05 (±3.12E-05)	6.95E-05 (±2.59E-05)	1.11E-04 (±5.95E-05)	5.78E-05 (±2.40E-05)
Aspartate	1.41E-03 (±3.52E-04)	1.29E-03 (±4.03E-04)	1.28E-03 (±3.03E-04)	1.14E-03 (±3.17E-04)

Sarcosine	1.33E-05 (±5.26E-06)	1.16E-05 (±4.30E-06)	1.43E-05 (±4.25E-06)	1.20E-05 (±3.95E-06)
2-Oxocaproate	9.24E-05 (±3.28E-05)	8.78E-05 (±2.40E-05)	1.14E-04 (±2.51E-05)	8.66E-05 (±3.58E-05)
Dimethylamine	1.73E-05 (±4.37E-06)	1.77E-05 (±6.16E-06)	2.34E-05 (±9.39E-06)	2.37E-05 (±1.18E-05)
Methionine	9.06E-04 (±1.50E-04)	9.59E-04 (±2.28E-04)	8.88E-04 (±9.30E-05)	8.07E-04 (±1.34E-04)
2-Oxoisocaproate	1.37E-04 (±4.64E-05)	1.17E-04 (±5.32E-05)	1.15E-04 (±5.49E-05)	1.11E-04 (±3.60E-05)
Methylamine	1.28E-04 (±4.63E-05)	1.42E-04 (±5.66E-05)	1.47E-04 (±3.55E-05)	1.63E-04 (±3.77E-05)
B-Alanine	2.53E-04 (±1.39E-04)	1.55E-04 (±5.12E-05)	2.62E-04 (±1.74E-04)	1.96E-04 (±1.30E-04)
Succinate	6.54E-04 (±2.85E-04)	5.55E-04 (±2.99E-04)	3.49E-04 (±1.47E-04)	2.46E-03 (±2.14E-03)
Pyruvate	6.52E-05 (±2.57E-05)	7.07E-05 (±3.52E-05)	5.99E-05 (±1.53E-05)	5.74E-05 (±2.78E-05)
Glutamate	3.54E-03 (±7.56E-04)	4.23E-03 (±8.42E-04)	3.64E-03 (±5.74E-04)	4.03E-03 (±1.38E-03)
Acetoacetate	1.79E-04 (±4.75E-05)	2.71E-04 (±1.26E-04)	1.72E-04 (±5.53E-05)	2.73E-04 (±1.47E-04)
5-Aminopentanoate	9.59E-04 (±2.43E-04)	9.22E-04 (±2.24E-04)	1.13E-03 (±2.24E-04)	1.04E-03 (±6.74E-05)
Acetate	5.91E-02 (±2.18E-02)	5.42E-02 (±1.63E-02)	5.63E-02 (±1.91E-02)	7.55E-02 (±3.81E-02)
Alanine	5.99E-03 (±8.94E-04)	6.50E-03 (±1.16E-03)	4.73E-03 (±7.03E-04)	4.61E-03 (±1.17E-03)
Ethanol	4.23E-04 (±3.12E-04)	3.15E-04 (±1.56E-04)	4.14E-04 (±1.12E-04)	5.78E-04 (±2.59E-04)
3-Methyl-2-oxovalerate	1.98E-04 (±7.33E-05)	1.67E-04 (±6.50E-05)	1.60E-04 (±3.10E-05)	1.73E-04 (±1.02E-04)
Isobutyrate	5.89E-03 (±1.58E-03)	5.60E-03 (±1.89E-03)	6.10E-03 (±1.61E-03)	6.42E-03 (±1.05E-03)
Propionate	1.84E-02 (±4.24E-03)	1.76E-02 (±4.84E-03)	1.87E-02 (±3.03E-03)	2.31E-02 (±4.75E-03)
Isoleucine	1.72E-03 (±2.66E-04)	1.80E-03 (±4.46E-04)	1.51E-03 (±1.52E-04)	1.56E-03 (±2.51E-04)
Valine	2.60E-03 (±3.45E-04)	2.56E-03 (±5.76E-04)	2.12E-03 (±2.99E-04)	2.19E-03 (±3.47E-04)
Leucine	4.57E-03 (±8.91E-04)	4.65E-03 (±9.80E-04)	3.80E-03 (±4.16E-04)	3.97E-03 (±8.66E-04)
Isovalerate	1.32E-03 (±4.65E-04)	1.47E-03 (±6.79E-04)	1.39E-03 (±4.95E-04)	9.20E-04 (±3.99E-04)
Butyrate	1.37E-02 (±6.18E-03)	1.36E-02 (±7.44E-03)	9.41E-03 (±2.83E-03)	1.38E-02 (±9.15E-03)
Valerate	1.98E-03 (±1.07E-03)	1.69E-03 (±1.23E-03)	2.01E-03 (±1.10E-03)	2.24E-03 (±5.69E-04)

	NGI		GI	
T2	Placebo	Probiotic	Placebo	Probiotic
Nicotinate	1.92E-04 (±4.50E-05)	1.74E-04 (±3.60E-05)	1.83E-04 (±4.55E-05)	1.96E-04 (±4.30E-05)
Formate	1.10E-04 (±3.27E-05)	1.15E-04 (±2.12E-05)	1.51E-04 (±4.96E-05)	1.58E-04 (±5.20E-05)
Hypoxanthine	2.48E-04 (±9.60E-05)	2.08E-04 (±6.84E-05)	2.11E-04 (±8.60E-05)	3.50E-04 (±1.41E-04)
Xanthine	3.11E-04 (±1.02E-04)	2.48E-04 (±6.90E-05)	2.65E-04 (±8.10E-05)	2.72E-04 (±6.44E-05)
Tryptophan	2.95E-04 (±2.40E-05)	2.61E-04 (±1.50E-05)	3.15E-04 (±2.95E-05)	2.80E-04 (±1.73E-05)
Phenylalanine	1.28E-03 (±1.90E-04)	1.41E-03 (±1.30E-04)	1.24E-03 (±1.75E-04)	1.21E-03 (±1.06E-04)
Phenylacetate	9.02E-04 (±3.58E-04)	1.01E-03 (±3.20E-04)	1.16E-03 (±4.59E-04)	5.28E-04 (±3.30E-04)
3-Hydroxyphenylacetate	1.97E-04 (±8.90E-05)	1.76E-04 (±7.00E-05)	1.22E-04 (±2.18E-05)	2.53E-04 (±1.05E-04)
Tyramine	6.76E-05 (±4.51E-05)	7.21E-05 (±3.59E-05)	3.93E-05 (±2.72E-05)	1.65E-04 (±7.80E-05)
Tyrosine	2.78E-03 (±2.40E-04)	2.90E-03 (±2.20E-04)	2.78E-03 (±1.60E-04)	2.31E-03 (±2.80E-04)
4-Hydroxyphenylacetate	1.02E-04 (±3.08E-05)	9.62E-05 (±2.67E-05)	1.09E-04 (±2.79E-05)	1.22E-04 (±3.78E-05)
pCresol	7.83E-05 (±5.52E-05)	3.66E-05 (±1.54E-05)	4.50E-05 (±2.52E-05)	5.64E-05 (±2.47E-05)
Fumarate	5.35E-04 (±1.78E-04)	5.15E-04 (±2.57E-04)	1.79E-04 (±9.30E-05)	2.54E-04 (±1.07E-04)
Orotate	5.44E-05 (±1.82E-05)	6.33E-05 (±2.05E-05)	6.37E-05 (±1.19E-05)	7.09E-05 (±2.24E-05)
Uridine	5.07E-05 (±1.82E-05)	5.05E-05 (±1.63E-05)	7.85E-05 (±1.97E-05)	6.00E-05 (±1.79E-05)
Uracil	8.98E-04 (±2.59E-04)	8.31E-04 (±1.70E-04)	7.50E-04 (±2.44E-04)	7.66E-04 (±2.41E-04)
Galactose	1.56E-04 (±5.10E-05)	1.41E-04 (±2.40E-05)	1.18E-04 (±2.47E-05)	2.18E-04 (±1.22E-04)
Xylose	1.62E-04 (±8.30E-05)	9.18E-05 (±5.48E-05)	7.57E-05 (±3.84E-05)	1.68E-04 (±7.70E-05)
Fucose	9.91E-05 (±1.86E-05)	1.26E-04 (±5.14E-05)	9.97E-05 (±1.84E-05)	1.65E-04 (±4.10E-05)

Arabinose	3.03E-04 (±8.50E-05)	2.34E-04 (±8.60E-05)	2.33E-04 (±9.80E-05)	4.98E-04 (±2.45E-04)
1.3-Dihydroxyacetone	1.63E-04 (±3.00E-05)	1.87E-04 (±7.30E-05)	1.09E-04 (±4.63E-05)	2.32E-04 (±5.55E-05)
Threonine	1.89E-03 (±1.70E-04)	2.24E-03 (±2.00E-04)	1.76E-03 (±3.95E-04)	1.91E-03 (±2.70E-04)
Serine	1.19E-03 (±2.03E-04)	1.41E-03 (±1.70E-04)	1.42E-03 (±2.45E-04)	1.30E-03 (±9.00E-05)
Glycerol	4.02E-04 (±1.27E-04)	3.01E-04 (±1.67E-04)	3.30E-04 (±2.25E-04)	7.82E-04 (±4.58E-04)
Glycine	1.88E-03 (±4.60E-04)	1.88E-03 (±3.44E-04)	1.69E-03 (±3.50E-04)	1.88E-03 (±4.10E-04)
Methanol	3.05E-04 (±8.70E-05)	2.10E-04 (±6.60E-05)	2.32E-04 (±4.25E-05)	3.29E-04 (±2.11E-04)
Proline	7.29E-04 (±3.37E-04)	8.47E-04 (±2.03E-04)	7.14E-04 (±2.01E-04)	9.19E-04 (±3.15E-04)
Glucose	4.82E-03 (±2.58E-03)	3.99E-03 (±2.67E-03)	4.38E-03 (±2.95E-03)	9.26E-03 (±5.04E-03)
Malonate	2.28E-03 (±6.40E-04)	2.10E-03 (±8.30E-04)	2.56E-03 (±1.30E-03)	2.00E-03 (±9.90E-04)
Creatine	1.05E-04 (±2.49E-05)	1.10E-04 (±1.70E-05)	1.02E-04 (±9.85E-06)	1.11E-04 (±3.52E-05)
N.N-Dimethylglycine	5.02E-05 (±3.22E-05)	6.42E-05 (±3.43E-05)	4.84E-05 (±2.75E-05)	3.82E-05 (±1.85E-05)
N-Methylhydantoin	3.05E-05 (±8.80E-06)	3.27E-05 (±1.33E-05)	3.84E-05 (±1.64E-05)	3.92E-05 (±1.37E-05)
TMA	1.05E-04 (±4.59E-05)	7.44E-05 (±2.76E-05)	9.07E-05 (±2.86E-05)	1.04E-04 (±2.10E-05)
Aspartate	9.92E-04 (±3.08E-04)	1.50E-03 (±5.00E-04)	1.41E-03 (±2.95E-04)	1.37E-03 (±4.10E-04)
Sarcosine	1.47E-05 (±5.45E-06)	1.24E-05 (±5.76E-06)	1.78E-05 (±5.50E-06)	1.05E-05 (±6.62E-06)
2-Oxocaproate	9.47E-05 (±3.88E-05)	8.44E-05 (±1.76E-05)	1.02E-04 (±3.11E-05)	5.75E-05 (±2.93E-05)
Dimethylamine	1.71E-05 (±1.10E-05)	2.10E-05 (±5.10E-06)	2.78E-05 (±1.86E-05)	1.72E-05 (±9.17E-06)
Methionine	8.87E-04 (±1.09E-04)	9.42E-04 (±1.08E-04)	9.00E-04 (±1.20E-04)	7.65E-04 (±7.70E-05)
2-Oxoisocaproate	9.35E-05 (±6.75E-05)	1.18E-04 (±4.30E-05)	1.49E-04 (±7.42E-05)	1.16E-04 (±6.39E-05)
Methylamine	1.84E-04 (±6.20E-05)	1.63E-04 (±3.60E-05)	1.98E-04 (±7.25E-05)	1.83E-04 (±3.16E-05)
B-Alanine	2.42E-04 (±1.11E-04)	1.86E-04 (±7.20E-05)	2.00E-04 (±4.35E-05)	1.72E-04 (±2.40E-05)
Succinate	6.91E-04 (±3.10E-04)	5.63E-04 (±2.63E-04)	4.49E-04 (±2.74E-04)	4.48E-04 (±1.39E-04)
Pyruvate	5.84E-05 (±2.72E-05)	5.97E-05 (±1.62E-05)	7.26E-05 (±7.10E-06)	6.24E-05 (±1.06E-05)
Glutamate	3.70E-03 (±9.20E-04)	4.21E-03 (±9.20E-04)	2.86E-03 (±9.20E-04)	4.07E-03 (±1.24E-03)
Acetoacetate	1.46E-04 (±4.10E-05)	1.84E-04 (±4.60E-05)	1.51E-04 (±3.25E-05)	2.09E-04 (±5.20E-05)
5-Aminopentanoate	1.06E-03 (±3.57E-04)	1.22E-03 (±3.82E-04)	9.24E-04 (±2.84E-04)	1.14E-03 (±1.84E-04)
Acetate	6.72E-02 (±1.60E-02)	5.13E-02 (±1.77E-02)	5.02E-02 (±1.49E-02)	7.64E-02 (±1.08E-02)
Alanine	5.60E-03 (±7.90E-04)	6.62E-03 (±7.30E-04)	5.48E-03 (±7.80E-04)	5.50E-03 (±7.50E-04)
Ethanol	5.26E-04 (±3.09E-04)	2.62E-04 (±1.18E-04)	3.20E-04 (±2.19E-04)	8.13E-04 (±5.97E-04)
3-Methyl-2-oxovalerate	2.02E-04 (±7.80E-05)	2.44E-04 (±9.90E-05)	1.94E-04 (±6.79E-05)	1.39E-04 (±2.20E-05)
Isobutyrate	6.62E-03 (±1.01E-03)	5.40E-03 (±1.34E-03)	6.94E-03 (±1.37E-03)	5.46E-03 (±2.57E-03)
Propionate	2.28E-02 (±4.10E-03)	1.80E-02 (±4.20E-03)	2.09E-02 (±6.40E-03)	1.74E-02 (±6.60E-03)
Isoleucine	1.67E-03 (±2.70E-04)	1.87E-03 (±1.76E-04)	1.80E-03 (±1.40E-04)	1.55E-03 (±2.10E-04)
Valine	2.49E-03 (±4.10E-04)	2.57E-03 (±2.30E-04)	2.50E-03 (±1.90E-04)	2.11E-03 (±2.60E-04)
Leucine	4.24E-03 (±8.70E-04)	4.72E-03 (±5.50E-04)	4.38E-03 (±5.45E-04)	3.64E-03 (±5.30E-04)
Isovalerate	1.22E-03 (±4.20E-04)	1.48E-03 (±5.00E-04)	1.48E-03 (±5.83E-04)	4.77E-04 (±1.97E-04)
Butyrate	1.63E-02 (±6.20E-03)	1.24E-02 (±4.77E-03)	1.17E-02 (±7.88E-03)	1.64E-02 (±1.04E-02)
Valerate	2.46E-03 (±1.25E-03)	1.39E-03 (±7.59E-04)	1.66E-03 (±8.61E-04)	1.78E-03 (±1.23E-03)
